# The moderating effect of parental skills for antibiotic identification on the link between parental skills for antibiotic use and inappropriate antibiotic use for children in China

**DOI:** 10.1186/s12889-023-15099-8

**Published:** 2023-01-23

**Authors:** Bo Yan, Zhenke He, Shixin Dong, Hailati Akezhuoli, Xin Xu, Xiaomin Wang, Xudong Zhou

**Affiliations:** Institute of Social Medicine, School of Medicine, Zhejiang University, Hangzhou, China

**Keywords:** Skill, Antibiotic identification, Antibiotic use, Self-medication, Children

## Abstract

**Background:**

Inappropriate antibiotic consumption promotes antibiotic resistance. However, findings on the association between antibiotic-related knowledge and behaviors are inconsistent and contradictory, resulting in unjustified guidance of interventions. The mechanisms between the different kinds of antibiotic-related skills contained in knowledge modules in some previous studies are indistinct and rarely studied.

**Methods:**

A cross-sectional survey was conducted between June 2017 and April 2018 in three Chinese provinces, investigating 9526 parents with children aged 0–13 years old. Data from 1944 parents who self-medicated their children and 2478 respondents whose children sought care were analyzed.

**Results:**

Skills for antibiotic identification were found to be a moderator for the association between skills for antibiotic use and two inappropriate behaviors. Compared with parents with low levels of both skills for antibiotic identification and use, those mastering both skills at either medium (OR = 0.48, 95% CI [0.26–0.88]) or high (OR = 0.15, 95% CI [0.07–0.34]) level were less likely to self-medicate their children with antibiotics. Parents with a medium level of skills for antibiotic identification and high level of skills for antibiotic use (OR = 0.18, 95% CI [0.08–0.44]) and those with a high level of both skills (OR = 0.15, 95% CI [0.05–0.47]) were less likely to ask doctors for antibiotics when seeking care.

**Conclusion:**

Parents’ high level of skills for antibiotic identification is revealed to promote inappropriate antibiotic use when parents master a low level of skills for antibiotic use. Conversely, based on excellent skills for antibiotic use, better skill for antibiotic identification is associated with a greater reduction in inappropriate behaviors. We recommend future health education to strengthen skills for antibiotic identification along with guidance on antibiotic use.

**Supplementary Information:**

The online version contains supplementary material available at 10.1186/s12889-023-15099-8.

## Introduction

Antimicrobial resistance (AMR) has been identified as one of the top ten global health threats by the World Health Organization (WHO) in 2019 [1], leading to high mortality and enormous financial loss [2]. A systematic analysis based on 204 countries and territories revealed that an estimated 4.95 million deaths were associated with bacterial AMR in 2019, of which approximately 1.27 million deaths were directly attributed to bacterial AMR [3]. Inappropriate antibiotics use among medical prescribers and patients promotes the development of AMR [4]. Self-medication with antibiotics and asking doctors for antibiotics when seeking care are major contributors to inappropriate antibiotic use among patients, especially in low- and middle-income countries (LMICs) [5–7]. The situation is particularly prominent among children and remains arduous to address [8].

A considerable body of research has studied the relationship between knowledge and behaviors of inappropriate antibiotic use [9], but with inconsistent conclusions. Studies from Jordan, Yemen, and Uzbekistan, for instance, suggested that patients with poorer knowledge were more likely to self-medicate with antibiotics and ask doctors for antibiotics when seeking care than those with better knowledge [10, 11]. Conversely, a study conducted among Chinese college students revealed that better knowledge was significantly associated with higher rates of self-medication with antibiotics [12]. The conflicting role of knowledge in predicting behaviors implied the possibility of inconsistent measurement of antibiotic use knowledge. Antibiotic use knowledge is usually measured by basic facts about antibiotics, awareness of AMR, potential harms of inappropriate antibiotic use, and skills for antibiotic use [13, 14]. However, these items were merely converted into fractions for simple addition, resulting in the complexity of the relationship between different knowledge modules and behaviors being invariably overlooked [15]. Particularly, the skills for antibiotic use, that is, whether antibiotics should be used in different scenarios, have been rarely studied. Many behavioral theories, including the Information, Motivation and Behavioral (IMB) Skills Model, Behavioral Change Wheel (BCW), and Behavioral Change Technical Taxonomy (BCTTv1), point out that skill is a significant inducement for behavior change, that is, knowledge and other factors achieve behavior change by improving related skills [16–18].

Among the antibiotic-related skills, skills for antibiotic identification have sometimes been neglected. Antibiotics have a variety of chemical names and trade names, which makes it difficult for the general public to identify them as antibiotics. In China, antibiotic drugs are called anti-inflammatory drugs with misconception or direct chemical names rather than antibiotics. A study on Chinese college students revealed that misinterpreting antibiotics as anti-inflammatory drugs significantly increased self-medication with antibiotics [19]. Similarly, research conducted in rural Ghana found that a lack of knowledge on the identification of antibiotics increased the risk of inappropriate antibiotic use [20]. These studies suggested that skills for antibiotic identification may play an important role in predicting inappropriate antibiotic use behaviors, however, few studies have examined their associations.

**Fig. 1:**
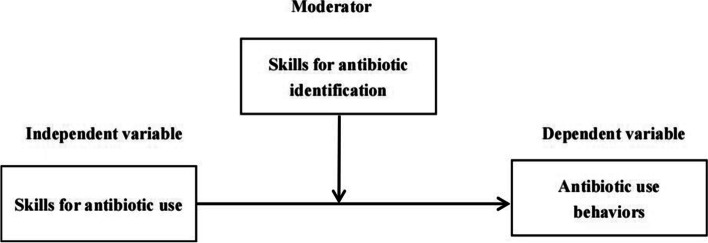
Moderation model

Consequently, this study aims to investigate the moderating effect of parental skills for antibiotic identification on the link between parental skills for antibiotic use and inappropriate antibiotic use for children in China (Fig. 1). Our hypothesis is that high level of parental skills for antibiotic identification will significantly exacerbate the influence of parental skills for antibiotic use on inappropriate antibiotic use behaviors.

## Methods

### Study design and participants

Data for this study came from a cross-sectional survey of parents with children aged 0–13 years in China conducted between June 2017 and April 2018. Three provinces representing different geographic regions and various levels of economic development were selected, including Zhejiang (East, ranked 5^th^ out of 31 provinces in terms of gross domestic product per capita in 2017), Shaanxi (Central-Northwest, ranked 12^th^), and Guangxi (Southwest, ranked 26^th^). A representative sample was acquired from the above provinces by implementing multistage stratified random cluster sampling, and successive stages of the sampling procedure included provinces, cities of prefecture level, cities, and rural areas in sequence. The sample population included all parents of children aged 3–13 who attended selected kindergartens and primary schools and all parents who brought their children under the age of 3 to the selected community health service center for routine physical examination and vaccination on weekdays. More specific sampling and recruitment processes have been reported in previous studies [8, 21, 22].

### Measurements

All recruited individuals completed a self-administered structured questionnaire, which was refined and finalized by applying the main findings from previous qualitative interviews and pilot testing based on the literature review. Three sections of the questionnaire were selected for this study: 1) Socio-demographic characteristics (i.e., parents’ gender, average monthly household income, education level, residence, medical education background, and their children’s age and gender); 2) Skills for antibiotic identification and skills for antibiotic use (Table A1); 3) Parental self-medication with antibiotics for their children and asking doctors for antibiotics when seeking care. Notably, the assessment of skills for antibiotic identification and antibiotic use incorporated grading scores of six and five questions, respectively (See supplementary data). The skills for identification were assessed by asking whether Amoxicillin, Cephalosporins, Non-steroidal anti-inflammatory drugs (NSAIDs), Steroids, Quinolones, and Macrolides are antibiotics. The skills for antibiotic use were evaluated by asking whether antibiotics should be used in the context of different self-limited symptoms such as fever, cough, and runny nose. 1 point was awarded if the answer was correct, whereas 0 point if participants made the wrong choice between "Yes" and "No", or choose "Don't know" directly.

The total scores of skills for antibiotic identification were grouped into three levels (low = 0–2 points, medium = 3–4 points, high = 5–6 points), and similarly, skills for antibiotic use were collapsed into three grades (low = 0–1 point, medium = 2–3 points, high = 4–5 points). Parental self-medication with antibiotics and asking doctors for antibiotics when seeking care were measured by whether the respondents (i) self-medicated their children with antibiotics when their children fell ill in the last month, (ii) asked doctors for antibiotics when seeking care for their children. In total, 1944 parents who had self-medicated their children and 2478 parents who had taken their children to the hospital in the past month were retrieved from the total of 9526 valid samples.

### Statistical analysis

**Fig. 2:**
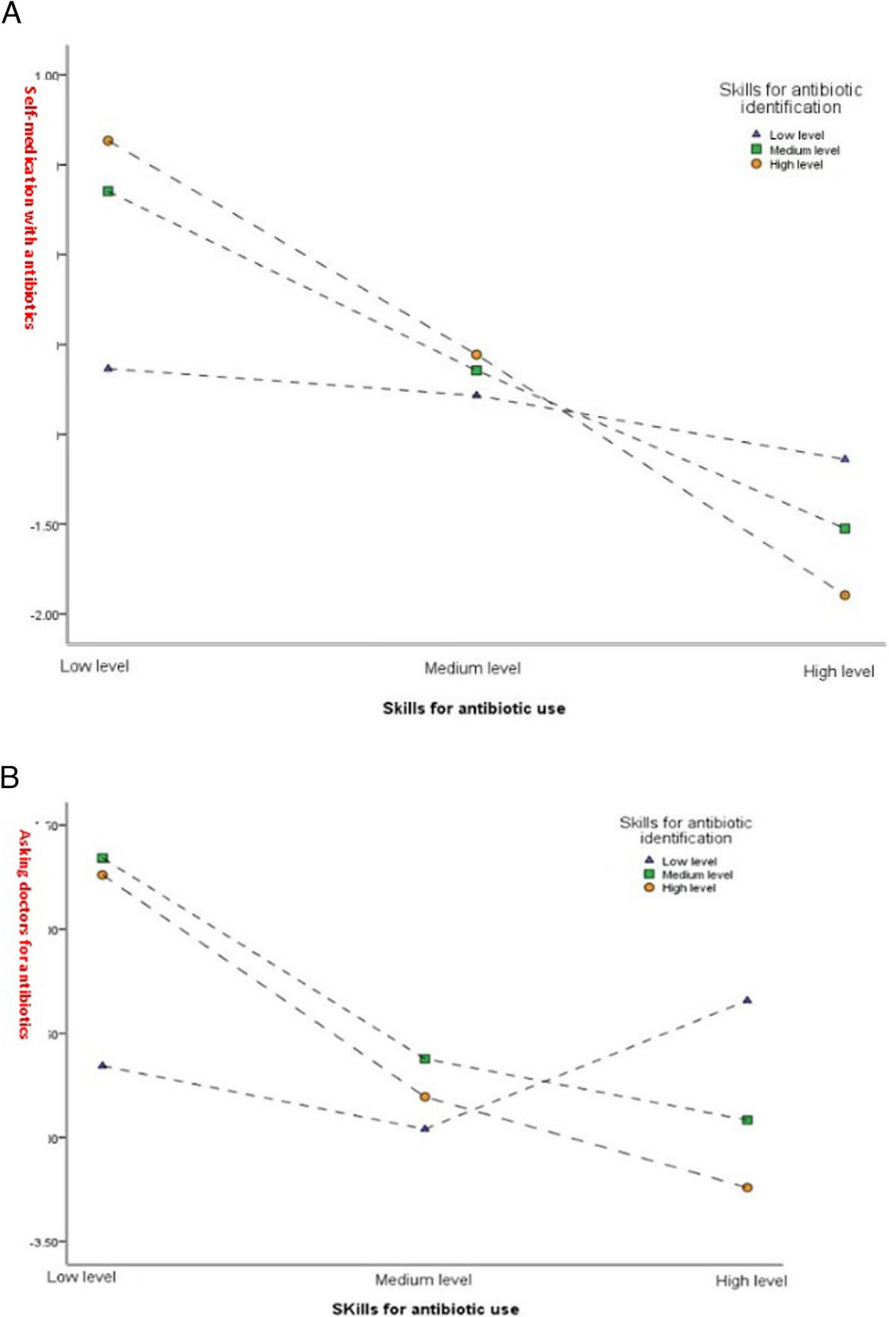
**a** Moderation of skills for antibiotic identification on the link between self-medication with antibiotics and skills for antibiotic use among parents who self-medicated their children(*N* = 1944). **b** Moderation of skills for antibiotic identification on the link between asking doctors for antibiotics when seeking care and skills for antibiotic use among parents whose children visited hospital(*N* = 2478)

Descriptive analyzes were performed to summarize the weighted frequencies and percentages of individual characteristics. Chi-square tests and t tests were performed to explore the correlation between socio-demographic characteristics and antibiotic-related skills and behaviors (Table A2) [23]. With the control of socio-demographic characteristics, binary logistic regression models were adopted to examine the effect of the two skills on the two inappropriate antibiotic use behaviors including self-medication with antibiotics and asking doctors for antibiotics when seeking care separately [24]. Influences of skills for antibiotic identification on behaviors were conducted in model 1, and model 2 revealed the influences of skills for antibiotic use on behaviors. The combined effect of two skills on behaviors was shown in model 3. In model 4, the interaction between skills regarding the two behaviors, which is equivalent to the moderating effect of skills for antibiotic identification on the association between skills for antibiotic use and two behaviors, was added for analysis with binary logistic regression and Process Model 1 (Fig. 2) [25]. Gamma correlation coefficient was used to test the association between the two skills (Tables A3 & A4). In testing the moderating effect, which is robust to our level-ranking of skills for antibiotic identification and use, the study analysis repeated the same operation as a sensitivity analysis based on treating the independent variables as continuous variables (Table A5). Full details can be accessed in the supplementary data. All statistical analyses were performed using SPSS version 27.0, based on a statistical significance level of *p* < 0.05.

## Results

### Basic information of the sample

As presented in Table 1, 1944 parents (559 from Zhejiang, 689 from Guangxi, 696 from Shaanxi) had self-medicated their children, and 2478 respondents (716 from Zhejiang, 863 from Guangxi, 899 from Shaanxi) had sought care in the previous month. Of all the children who were self-medicated, nearly half were girls (48.6%), with an average age of 5.5 years (SD = 3.1). 53.8% of them had a parent with a junior college and above educational level and 14.2% of them had a parent with a medical background. 41.3% of respondents lived in rural areas; half of them had a monthly average household income under 5000 RMB (US$769); and nearly 80% had a medium or high level of skill for antibiotic identification or use, separately. Among those who sought care, there were slightly more male children than females (1274, 51.4%, vs 1204, 48.6%), and their mean age was 5.2 years (SD = 3.3). Less than half of the parents (47.3%) had college and above levels of education. Only 10.5% of the respondents had a medical background, 47.2% of respondents’ monthly average household income was over 5000 RMB (US$769), and 55.8% were urban residents. About 70% of parents mastered a medium or high level of skill for antibiotic identification or use.

**Table 1:** Socio-demographic characteristics of children and parents stratified by different behaviors

Characteristics	Self-medication(*N* = 1944), N(%)^a^					Hospital visit(*N* = 2478), N(%)^a^				
	total	With antibiotics	Without antibiotics	χ^2^ / t	*P* value	total	Antibiotic requirement	No antibiotic requirement	χ^2^ / t	*P* value
Province				81.391	< 0.001				1.073	0.585
Zhejiang	559(28.8)	105(18.8)	454(81.2)			716(28.9)	56(7.8)	660(92.2)		
Guangxi	689(35.4)	219(31.8)	470(68.2)			863(34.8)	69(8.0)	794(92.0)		
Shaanxi	696(35.8)	297(42.7)	399(57.3)			899(36.3)	61(6.8)	838(93.2)		
Gender				0.197	0.657				1.354	0.245
Male	1000(51.4)	324(32.4)	676(67.6)			1274(51.4)	88(6.9)	1186(93.1)		
Female	944(48.6)	297(31.5)	647(68.5)			1204(48.6)	98(8.1)	1106(91.9)		
Age(years), Mean(SD)	5.5(3.1)	5.6(3.0)	5.4(3.1)	1.825	0.068	5.2(3.3)	6.3(3.3)	5.1(3.3)	4.685	< 0.001
Parents’ highest level of education				18.822	< 0.001				16.207	< 0.001
Middle school and under	382(19.7)	149(39.0)	233(61.0)			604(24.4)	68(11.2)	538(88.8)		
High school	515(26.5)	180(35.0)	335(65.0)			701(28.3)	47(6.7)	654(93.3)		
Junior college and above	1047(53.8)	292(27.9)	755(72.1)			1171(47.3)	71(6.1)	1100(93.9)		
Residence				2.140	0.143				0.795	0.373
Rural	802(41.3)	271(33.8)	531(66.2)			1095(44.2)	88(8.0)	1007(92.0)		
Urban	1142(58.7)	350(30.6)	792(69.4)			1383(55.8)	98(7.1)	1285(92.9)		
Average household income (RMB, monthly)				41.057	< 0.001				5.392	0.145
< = 3000 (US$461)	374(19.2)	146(39.0)	228(61.0)			511(20.6)	50(9.8)	461(90.2)		
3001–5000 (US$462–769)	592(30.5)	227(38.3)	365(61.7)			797(32.2)	58(7.3)	739(92.7)		
5001–10,000 (US$770–1538)	586(30.1)	158(27.0)	428(73.0)			742(29.9)	47(6.3)	695(93.7)		
> = 10,001 (US$1538)	392(20.2)	90(23.0)	302(77.0)			428(17.3)	31(7.2)	397(92.8)		
Parents with medical background				1.743	0.187				0.012	0.913
Yes	277(14.2))	79(28.5)	198(71.5)			259(10.5)	19(7.3)	240(92.7)		
No	1667(85.8)	542(32.5)	1125(67.5)			2219(89.5)	167(7.5)	2052(92.5)		
Skills for antibiotic identification				3.107	0.212				3.957	0.138
Low	435(22.4)	132(30.3)	303(69.7)			702(28.3)	47(6.7)	655(93.3)		
Medium	964(49.6)	326(33.8)	638(66.2)			1187(47.9)	102(8.6)	1085(91.4)		
High	545(28.0)	163(29.9)	382(70.1)			589(23.8)	37(6.3)	552(93.7)		
Skills for antibiotic use				144.862	< 0.001				24.544	< 0.001
Low	429(22.1)	220(51.3)	209(48.7)			692(27.9)	81(11.7)	611(88.3)		
Medium	808(41.6)	278(34.4)	530(65.6)			983(39.7)	60(6.1)	923(93.9)		
High	707(36.3)	123(17.4)	584(82.6)			803(32.4)	45(5.6)	758(94.4)		

*SD* standard deviation

^a^Data are N(%) unless otherwise stated

Significant differences were found in terms of the province, the highest level of education, and average household income between parents who self-medicated their children with antibiotics and those without antibiotics (*p* < 0.001). Parents with higher education attainment, higher income, and who settled in Zhejiang province had a lower probability of engaging in self-medication. Similarly, noteworthy differences were revealed regarding age and the highest level of education between respondents who asked doctors for antibiotics when seeking care and those who did not (*p* < 0.001). Highly educated parents and those whose children are younger were less inclined to asking doctors for antibiotics when seeking care. Besides, the rates of self-medication with antibiotics and asking doctors for antibiotics when seeking care were both significantly lower in the respondents with a higher level of skills for antibiotic use (*p* < 0.001).

### Association between antibiotic-related skills and inappropriate behaviors

In adjusted models (Tables 2 and 3), after controlling for socio-demographic characteristics, for self-medication with antibiotics, model 1 showed that respondents with a medium level of skills for antibiotic identification (aOR = 1.36, 95% CI [1.05–1.77]) had prominently higher odds of self-medication with antibiotics for their children. Model 2 showed that parents with medium (aOR = 0.54, 95% CI [0.42–0.69]) and high (aOR = 0.23, 95% CI [0.17–0.31]) levels of skill for antibiotics use were less likely to conduct self-medication with antibiotics for their children. For asking doctors for antibiotics when seeking care, model 1 exhibited that it was not associated with skills for antibiotic identification after adjusting for socio-demographic characteristics. In model 2, parents mastering medium and higher levels of skills for antibiotic use were less likely (aOR = 0.51, 95% CI [0.35–0.73]; aOR = 0.47, 95% CI [0.32–0.71]) to ask doctors for antibiotics when seeking care.

**Table 2:** The interaction between skills for antibiotic identification and self-medication with antibiotics among parents who self-medicated their children(*N* = 1944)

	Model 1	Model 2	Model 3	Model 4
	aOR(95% CI)	aOR(95% CI)	aOR(95% CI)	aOR(95% CI)
Skills for antibiotic identification				
Low	Ref		Ref	Ref
Medium	1.36 (1.05,1.77)^*^		1.48 (1.13,1.94)^**^	2.68 (1.70,4.21)^***^
High	1.22 (0.90,1.65)		1.42 (1.03,1.95)^*^	3.67 (2.00,6.74)^***^
Skills for antibiotic use				
Low		Ref	Ref	Ref
Medium		0.54 (0.42,0.69)^***^	0.51 (0.40,0.66)^***^	0.88 (0.54,1.45)
High		0.23 (0.17,0.31)^***^	0.22 (0.17,0.30)^***^	0.64 (0.37,1.09)
Level of skills for identification × Level of skills for use				
Low Identification level × Low Using level				Ref
Medium Identification level × Medium Using level				0.48 (0.26,0.88)^*^
High Identification level × Medium Using level				0.36 (0.17,0.76)^**^
Medium Identification level × High Using level				0.29 (0.15,0.56)^***^
High Identification level × High Using level				0.15 (0.07,0.34)^***^

*aOR* adjusted odds ratio, *Ref* reference group

^*^ < 0.05; ** < 0.01; *** < 0.001

**Table 3:** The interaction between skills for antibiotic identification and asking doctors for antibiotics when seeking care among parents whose children visited hospital(*N* = 2478)

	Model 1	Model 2	Model 3	Model 4
	aOR(95% CI)	aOR(95% CI)	aOR(95% CI)	aOR(95% CI)
Skills for antibiotic identification				
Low	Ref		Ref	Ref
Medium	1.45 (1.00,2.12)		1.66 (1.13,2.45)^*^	3.21 (1.81,5.70)^***^
High	1.05 (0.65,1.69)		1.28 (0.78,2.08)	2.74 (1.26,5.96)^*^
Skills for antibiotic use				
Low		Ref	Ref	Ref
Medium		0.51 (0.35,0.73)^***^	0.48 (0.33,0.69)^***^	0.86 (0.39,1.90)
High		0.47 (0.32,0.71)^***^	0.46 (0.31,0.69)^***^	1.59 (0.80,3.18)
Level of skills for identification × Level of skills for use				
Low Identification level × Low Using level				Ref
Medium Identification level × Medium Using level				0.43 (0.17,1.09)
High Identification level × Medium Using level				0.42 (0.14,1.31)
Medium Identification level × High Using level				0.18 (0.08,0.44)^***^
High Identification level × High Using level				0.15 (0.05,0.47)^***^

*aOR* adjusted odds ratio, *Ref* reference group

^*^ < 0.05; ** < 0.01; *** < 0.001

Among 1944 parents who self-medicated their children in the previous month (Table 2), model 4 reveals that compared with parents with low level of both skills for antibiotic identification and use, parents with other different combinations of skill levels were all less likely to self-medicated their children, including those with medium level of both skills (OR = 0.48, 95% CI [0.26–0.88]), high level of skills for antibiotic identification and medium level of skills for antibiotic use (OR = 0.36, 95% CI [0.17–0.76]), medium level of skills for antibiotic identification and high level of skills for antibiotic use (OR = 0.29, 95% CI [0.15–0.56]) and high level of both skills (OR = 0.15, 95% CI [0.07–0.34]). That is, the effects of skills for antibiotic use on self-medication with antibiotics were moderated by skills for antibiotic identification (*P* < 0.05). As is shown in Fig. 2(a), for parents with equally low levels of skills for antibiotic use, higher levels of skills for antibiotic identification result in a greater likelihood of self-medication with antibiotics. In contrast, for parents with equally high levels of skills for antibiotic use, higher levels of skills for antibiotic identification were less likely to lead to self-medication with antibiotics. Such contrasts intuitively indicate the moderating effect of skills for antibiotic identification.

As is displayed in Table 3, the interaction of the two skills was negatively correlated with asking doctors for antibiotics when seeking care. Specifically, when compared with those with a low level of both skills, parents with a medium level of skill for antibiotic identification and a high level of skill for antibiotic use had a lower chance of asking doctors for antibiotics when seeking care (OR = 0.18, 95% CI [0.08–0.44]), and parents with a high level of both skills were also less inclined to asking doctors for antibiotics when seeking care (OR = 0.15, 95% CI [0.05–0.47]). As both skill levels rise, the likelihood of inappropriate behavior decreases, although not all categories of skill level were statistically significant. The trend in Fig. 2(b) further confirms this result. Similar to self-medication with antibiotics, when the level of skills for antibiotic use is low, the higher level of skills for antibiotic identification is accompanied by a higher likelihood of asking doctors for antibiotics when seeking care. But as the skill for antibiotic use is at a high level, the probability of asking doctors for antibiotics when seeking care of those parents with a higher level of skills for antibiotic identification becomes the lowest.

The results of the sensitivity analysis, treating the independent variables as continuous variables, were consistent with the above-mentioned conclusion, suggesting that the moderation is robust to the classification of skills for antibiotic identification and use.

## Discussion

To our knowledge, this is the first study to explore the moderating effect of skills for antibiotic identification on the association between skills for antibiotic use and inappropriate antibiotic use behaviors. Our results revealed that the correlation between skills for antibiotic use and inappropriate antibiotic use behaviors including self-medication with antibiotics and asking doctors for antibiotics when seeking care was moderated by skills for antibiotic identification. When the skills for antibiotic use are poor, the higher the level of skills for antibiotic identification, the more inappropriate use behaviors occur instead. Only parents who excelled in both skills were least likely to engage in inappropriate behaviors.

There are global efforts to reduce the inappropriate use of antibiotics through national or regional campaigns. However, studies have shown that the evidence base for many community-based antimicrobial stewardship (AMS) interventions is limited and heterogeneous, and existing cases failed to provide strong evidence to support well-established interventions [9, 26]. For example, a campaign conducted in England in 2008 with a key message of ‘The best way to treat most colds, coughs or sore throats is plenty of fluids and rest. For advice talk to your pharmacist or doctor’ [27] was revealed that there was little evidence that it worked. Researchers found little recollection of the campaign materials and no improvement in antibiotic use or attitudes through surveying the public who used to participate in the campaign. And it was suggested by the researchers that the government should reconsider the main messages of the interventions and materials used and conduct a procedural assessment of future campaigns, considering the relatively shallow material content of this campaign [28]. Indeed, the evidence suggests that education should be a core component of AMS, but the content of educational interventions needs to be carefully selected for what is truly effective [9].

In recent years, there has been a growing call to draw on theories, frameworks, and methods from the behavioral and social sciences to assess how AMS and interventions impact in an effective way. Many behavioral theories view skills as important triggers for behavior change [16–18] and have been successfully introduced for intervention. For example, a pilot study in Ghana aimed at raising parents' awareness of antibiotic resistance produced an animation based on IMB Skills Model as an intervention method, which achieved certain positive effects [29]. According to the IMB skills model, being knowledgeable and motivated can help individuals develop more adequate skills, which in turn can lead to better health behaviors [30]. So it can be seen that education on antibiotic use should focus on strengthening the development of skills.

Among antibiotic-related skills including antibiotic identification and use, skills for antibiotic identification are easy to be overlooked particularly. In China, the public's misconception is that antibiotics are equivalent to anti-inflammatory drugs, and which drugs belong to antibiotics largely depends on the experience of the masses rather than scientific guidance [19, 31]. Similarly, barriers to correctly identifying antibiotics have been revealed in many LMICs [32], especially in less educated areas. The habitual consciousness of the public is that knowing how to use antibiotics is enough to alleviate AMR. However, knowledge of antibiotics identification has been shown to help clear up some misconceptions [33]. Our previous findings on antibiotic misconceptions also confirm that misidentification is associated with an increase in inappropriate medication behaviors [19].

Our findings of the moderation effect in this study further reveal the significance of skills for antibiotic identification. For the group with poor skills for antibiotic use, the stronger the skills for antibiotic identification, the more likely the inappropriate behaviors are to occur, likely because although patients clearly know which drugs are antibiotics and effective for their symptoms, they do not know how to use them properly, which leads to unreasonable behaviors such as self-medication with antibiotics. On the contrary, based on poor skills for antibiotic identification, improvements in skills for antibiotic use are often accompanied by increases in inappropriate behaviors. Although patients are able to use drugs correctly, they are unable to identify whether the drugs used are antibiotics or not, also leading to the abuse of antibiotics. Therefore, to solve the problem of self-medication with antibiotics and asking doctors for antibiotics when seeking care, skills for antibiotic identification can no longer be ignored and we propose promoting education on antibiotic identification nationwide in the future.

Our findings have several implications for future policies and interventions. From the supply-side perspective, the findings give some directional recommendations on the need to strengthen the simultaneous education of people's multifaceted skills, especially the skills for antibiotic identification. For six consecutive years, the Chinese government has held the "Antimicrobial Awareness Week" in conjunction with the World Health Organization, which is a good opportunity to improve the antibiotic-related skills of the public [34]. Antibiotics should be clearly differentiated from other commonly marketed drugs, and clear antibiotic labels can make people better aware of their own antibiotic use [32]. A template worth learning came from India's nationwide voluntary antibiotic resistance awareness campaign, which mandates the display of red vertical bands on antibiotic packaging as a warning [35]. Similarly, the Chinese government can indicate whether the drug is an antibiotic on drug packaging, and antibiotic identification campaigns included in the daily activities of guidance for rational drug use in schools and communities through a variety of media forms were recommended. In addition, barriers in doctor-patient communication often lead to a stronger willingness for doctors to use antibiotics in patients [36]. Patients and their caregivers often challenge doctors’ authority by rejecting treatment recommendations and such pressure may increase physician prescribing behavior [37, 38]. In order to reduce the situation of patients actively asking doctors for antibiotics when seeking care, medical staff need to be trained to strengthen communication skills to be more adept at responding to the various demands and challenges of patients and it might be a fruitful choice to provide some suggestions on skills for antibiotic identification and use from doctors to patients. From the patients’ perspective, future interventions targeting the development of skills would help reduce inappropriate antibiotic use in both community and clinical settings. The public should actively correct their misconceptions and strengthen their ability to accurately identify antibiotics.

### Limitations

Some limitations should be noted. First, our study relied on parental self-reports, which could lead to recall bias. However, we tried to control this bias by asking parents about their behaviors and children’s symptoms within the recent month rather than longer. Second, the measurement of skills for antibiotic identification only included six questions, which might not be comprehensive enough. Third, our study only enrolled subjects whose parents were primary caregivers, excluding grandparents and other relatives. A children-based study reported that grandparents might be more likely than parents to self-medicate their children with antibiotics empirically in central and eastern China [39], thus the inappropriate antibiotic use behavior in our study might be underestimated.

## Conclusions

Within parental context, skills for antibiotic identification and use are of equal importance to the appropriate use of antibiotics, and are associated with behaviors such as self-medication with antibiotics and asking doctors for antibiotics when seeking care. Skills for antibiotic identification can moderate the association between skills for antibiotic use and behaviors, resulting in a directional change in the correlation. Skill-based tailored educational interventions need to be developed to reduce parental self-medication and demanding behaviors.

## Supplementary Information

**Additional file 1: Appendix. Table A1**. Specific questionnaire items. **Table A2.** (a)Association between socio-demographic characteristics and parents’ skills for antibiotic identification and antibiotic use among parents who self-medicated their children(*N*=1944). (b)Association between sociodemographic characteristics and parents’ skills for antibiotic identification and antibiotic use among parents whose children visited hospital(*N*=2478). **Table A3.** Correlation analysis between skills for antibiotic identification and skills for antibiotic use among parents who self-medicated their children(*N*=1944). **Table A4.** Correlation analysis between skills for antibiotic identification and skills for antibiotic use among parents whose children visited hospital(*N*=2478). **Table A5.** Sensitivity analysis. (a) Parents who self-medicated their children(*N*=1944) (b) Parents whose children visited hospital(*N*=2478).

## Data Availability

The datasets used and/or analyzed during the current study are available from the corresponding author on reasonable request.

## Abbreviations

AMR: Antimicrobial resistance
LMICs: Low- and middle- income countries
SD: Standard deviation
OR: Odds ratio
aOR: Adjusted odds ratio

## References

[1] Organization, W. H. Ten threats to global health in 2019. 2019. https://www.who.int/vietnam/news/feature-stories/detail/ten-threats-to-global-health-in-2019.

[2] Fu P, Xu H, Jing C (2021). Bacterial epidemiology and antimicrobial resistance profiles in children reported by the ISPED Program in China, 2016 to 2020. Microbiol Spectr.

[3] Antimicrobial Resistance Collaborators (2022). Global burden of bacterial antimicrobial resistance in 2019: a systematic analysis. Lancet.

[4] Organization WH (2001). Who global strategy for containment of antimicrobial resistance. Wkly Epidemiol Rec.

[5] Aslam A, Gajdács M, Zin CS (2020). Evidence of the practice of self-medication with antibiotics among the Lay Public in Low- and Middle-Income Countries: a scoping review. Antibiotics (Basel).

[6] Yu M, Zhao G, Stalsby Lundborg C (2014). Knowledge, attitudes, and practices of parents in rural China on the use of antibiotics in children: a cross-sectional study. BMC Infect Dis.

[7] Ong S, Moran GJ, Krishnadasan A (2011). Antibiotic prescribing practices of emergency physicians and patient expectations for uncomplicated lacerations. West J Emerg Med.

[8] Sun C, Hu YJ, Wang X (2019). Influence of leftover antibiotics on self-medication with antibiotics for children: a cross-sectional study from three Chinese provinces. BMJ Open.

[9] Lam TT, Dang DA, Tran HH (2021). What are the most effective community-based antimicrobial stewardship interventions in low- and middle-income countries? A narrative review. J Antimicrob Chemother.

[10] Shehadeh M, Suaifan G, Darwish RM (2012). Knowledge, attitudes and behavior regarding antibiotics use and misuse among adults in the community of Jordan. A pilot study Saudi Pharm J.

[11] Belkina T, Al Warafi A, Hussein Eltom E (2014). Antibiotic use and knowledge in the community of Yemen, Saudi Arabia, and Uzbekistan. J Infect Dev Ctries.

[12] Pan H, Cui B, Zhang D (2012). Prior knowledge, older age, and higher allowance are risk factors for self-medication with antibiotics among university students in southern China. PLoS One.

[13] Waaseth M, Adan A, Røen IL (2019). Knowledge of antibiotics and antibiotic resistance among Norwegian pharmacy customers - a cross-sectional study. BMC Public Health.

[14] Chang J, Lv B, Zhu S (2018). Non-prescription use of antibiotics among children in urban China: a cross-sectional survey of knowledge, attitudes, and practices. Expert Rev Anti Infect Ther.

[15] Sun R, Yao T, Zhou X (2022). Non-biomedical factors affecting antibiotic use in the community: a mixed-methods systematic review and meta-analysis. Clin Microbiol Infect.

[16] Michie S, Atkins L, West R (2014). The behaviour change wheel: a guide to designing interventions.

[17] Michie S, Richardson M, Johnston M (2013). The behavior change technique taxonomy (v1) of 93 hierarchically clustered techniques: building an international consensus for the reporting of behavior change interventions. Ann Behav Med.

[18] Goodell LS, Pierce MB, Amico KR (2012). Parental information, motivation, and behavioral skills correlate with child sweetened beverage consumption. J Nutr Educ Behav.

[19] Wang W, Wang X, Hu YJ (2019). The misconception of antibiotic equal to an anti-inflammatory drug promoting antibiotic misuse among Chinese University Students. Int J Environ Res Public Health.

[20] Afari-Asiedu S, Hulscher M, Abdulai MA (2020). Every medicine is medicine; exploring inappropriate antibiotic use at the community level in rural Ghana. BMC Public Health.

[21] Xu J, Wang X, Sun KS (2020). Parental self-medication with antibiotics for children promotes antibiotic over-prescribing in clinical settings in China. Antimicrob Resist Infect Control.

[22] Lin L, Harbarth S, Wang X (2020). Survey of parental use of antimicrobial drugs for common childhood infections, China. Emerg Infect Dis.

[23] Lee SW (2022). Methods for testing statistical differences between groups in medical research: statistical standard and guideline of Life Cycle Committee. Life Cycle.

[24] Lee SW (2022). Regression analysis for continuous independent variables in medical research: statistical standard and guideline of Life Cycle Committee. Life Cycle.

[25] Bolin JH, Hayes AF. Introduction to Mediation, Moderation, and Conditional Process Analysis: A Regression-Based Approach. New York: The Guilford Press. J Educ Meas. 2014;51:335–7.

[26] Fletcher-Miles H, Gammon J, Williams S (2020). A scoping review to assess the impact of public education campaigns to affect behavior change pertaining to antimicrobial resistance. Am J Infect Control.

[27] Gov.UK. Department of Health Antibiotic Campaign 2008–2009. 2009. http://www.dh.gov.uk/en/Publichealth/Patientsafety/Antibioticresistance/DH_082512.

[28] McNulty CA, Nichols T, Boyle PJ (2010). The English antibiotic awareness campaigns: did they change the public's knowledge of and attitudes to antibiotic use?. J Antimicrob Chemother.

[29] Appiah B, Asamoah-Akuoko L, Samman E (2022). The impact of antimicrobial resistance awareness interventions involving schoolchildren, development of an animation and parents engagements: a pilot study. Antimicrob Resist Infect Control.

[30] Fisher JD, Fisher WA, Misovich SJ (1996). Changing AIDS risk behavior: effects of an intervention emphasizing AIDS risk reduction information, motivation, and behavioral skills in a college student population. Health Psychol.

[31] Cabral C, Horwood J, Hay AD (2014). How communication affects prescription decisions in consultations for acute illness in children: a systematic review and meta-ethnography. BMC Fam Pract.

[32] Do NTT, Vu HTL, Nguyen CTK (2021). Community-based antibiotic access and use in six low-income and middle-income countries: a mixed-method approach. Lancet Glob Health.

[33] Ye L, Mao L. A study on effect of accompanied education on cognition, behavior of college students towards antibiotic application. Chin Nurs Res. 2005;19(11):2373–74.

[34] Department of Medical Administration. ‘Antimicrobial Awareness Week 2021’. 2021.http://www.nhc.gov.cn/yzygj/s3594/202111/91981fd3d3084accbd79b57309a134d8.shtml.

[35] Tamhankar AJ, Nachimuthu R, Singh R (2019). Characteristics of a Nationwide Voluntary Antibiotic Resistance Awareness Campaign in India; Future Paths and Pointers for Resource Limited Settings/Low and Middle Income Countries. Int J Environ Res Public Health.

[36] Colliers A, Bombeke K, Philips H (2021). Antibiotic prescribing and doctor-patient communication during consultations for respiratory tract infections: a video observation study in out-of-hours primary care. Front Med (Lausanne).

[37] Sirota M, Round T, Samaranayaka S (2017). Expectations for antibiotics increase their prescribing: Causal evidence about localized impact. Health Psychol.

[38] Wang NC, Liu Y (2021). Going shopping or consulting in medical visits: Caregivers' roles in pediatric antibiotic prescribing in China. Soc Sci Med.

[39] Li R, Xiao F, Zheng X (2016). Antibiotic misuse among children with diarrhea in China: results from a national survey. PeerJ.

