# Supplementary material for: The moderating effect of parental skills for antibiotic identification on the link between parental skills for antibiotic use and inappropriate antibiotic use for children in China
Source: BMC Public Health. 2023 Jan 23;23:156. doi: 10.1186/s12889-023-15099-8 (PMC9872431; doi:10.1186/s12889-023-15099-8)
Supplement: Supplementary file 1 — Additional file 1: Appendix. Table A1. Specific questionnaire items. Table A2. (a)Association between socio-demographic characteristics and parents’ skills for antibiotic identification and antibiotic use among parents who self-medicated their children(N=1944). (b)Association between sociodemographic characteristics and parents’ skills for antibiotic identification and antibiotic use among parents whose children visited hospital(N=2478). Table A3. Correlation analysis between skills for antibiotic identification and skills for antibiotic use among parents who self-medicated their children(N=1944). Table A4. Correlation analysis between skills for antibiotic identification and skills for antibiotic use among parents whose children visited hospital(N=2478). Table A5. Sensitivity analysis. (a) Parents who self-medicated their children(N=1944) (b) Parents whose children visited hospital(N=2478). [file 12889_2023_15099_MOESM1_ESM.docx]

**Appendix**

**Table A1 Specific questionnaire items**

|  | **Questionnaire**  **(1) Yes (2) No (3) Do not know** |
| --- | --- |
| **Skills for antibiotic identification** | 1. Do you think the following medicines are antibiotics? (Penicillins such as amoxicillin) 2. Do you think the following medicines are antibiotics? ( Cephalosporins such as Cefaclor/ceftriaxone sodium) 3. Do you think the following medicines are antibiotics? (NSAIDs such as ibuprofen/merrill/aspirin) 4. Do you think the following medicines are antibiotics? (Steroids such as dexamethasone/prednisone) 5. Do you think the following medicines are antibiotics? (Quinolones such as ofloxacin/norfloxacin) 6. Do you think the following medicines are antibiotics? (Macrolides such as azithromycin/roxithromycin) |
| **Skills for antibiotic use** | 1. Do you prefer to use antibiotics when you:    1. have sore throat?    2. have a cough/common cold/runny nose?    3. have a diarrhoea?    4. have a fever?    5. have a tympanitis? |

Notes:

Skills for antibiotic identification (Antibiotic recognition level): Low (0-2 points), Medium (3-4 points), High (5-6 points).

Skills for antibiotic use (Antibiotic using level): Low (0-1 points), Medium (2-3 points), High (4-5 points).

**Table A2**

**(a)Association between sociodemographic characteristics and parents’ skills for antibiotic identification and antibiotic use among parents who self-medicated their children(N=1944) (b)Association between sociodemographic** **characteristics and parents’ skills for antibiotic identification and antibiotic use among parents whose children visited hospital(N=2478)**

| (a) |  | | | | |  | | | | |
| --- | --- | --- | --- | --- | --- | --- | --- | --- | --- | --- |
| Characteristics | Skills for antibiotic identification(N=1944), N(%)^a^ | | | | | Skills for antibiotic use(N=1944), N(%)^a^ | | | | |
|  | Low | Median | High | χ^2^ / F | P value | Low | Median | High | χ^2^ / F | P value |
| Province |  |  |  | 30.770 | <0.001 |  |  |  | 57.400 | <0.001 |
| Zhejiang | 85(15.2) | 288(51.5) | 186(33.3) |  |  | 70(12.5) | 231(41.3) | 258(46.2) |  |  |
| Guangxi | 181(26.3) | 347(50.3) | 161(23.4) |  |  | 172(25.0) | 275(39.9) | 242(35.1) |  |  |
| Shaanxi | 169(24.3) | 329(47.3) | 198(28.4) |  |  | 187(26.9) | 302(43.4) | 207(29.7) |  |  |
| Gender |  |  |  | 2.837 | 0.242 |  |  |  | 0.121 | 0.941 |
| Male | 209(20.9) | 510(51.0) | 281(28.1) |  |  | 222(22.2) | 418(41.8) | 360(36.0) |  |  |
| Female | 226(23.9) | 454(48.1) | 264(28.0) |  |  | 207(21.9) | 390(41.3) | 347(36.8) |  |  |
| Age(years), Mean(SD) | 4.9(3.5) | 5.6(3.0) | 5.7(2.9) | 9.385 | <0.001 | 5.6(3.3) | 5.6(3.1) | 5.3(3.0) | 2.540 | 0.079 |
| Parents’ highest level of education |  |  |  | 153.617 | <0.001 |  |  |  | 137.863 | <0.001 |
| Middle school and under | 162(42.4) | 156(40.8) | 64(16.8) |  |  | 151(39.5) | 149(39.0) | 82(21.5) |  |  |
| High school | 124(24.1) | 280(54.4) | 111(21.5) |  |  | 135(26.2) | 221(42.9) | 159(30.9) |  |  |
| Junior college and above | 149(14.2) | 528(50.4) | 370(35.4) |  |  | 143(13.7) | 438(41.8) | 466(44.5) |  |  |
| Residence |  |  |  | 25.924 | <0.001 |  |  |  | 17.820 | <0.001 |
| Rural | 220(27.4) | 394(49.1) | 188(23.5) |  |  | 213(26.5) | 327(40.8) | 262(32.7) |  |  |
| Urban | 215(18.8) | 570(49.9) | 357(31.3) |  |  | 216(18.9) | 481(42.1) | 445(39.0) |  |  |
| Average household income (RMB, monthly) |  |  |  | 66.514 | <0.001 |  |  |  | 88.463 | <0.001 |
| < =3000 (US$461) | 126(33.7) | 180(48.1) | 68(18.2) |  |  | 122(32.6) | 155(41.5) | 97(25.9) |  |  |
| 3001–5000 (US$462–769) | 145(24.5) | 287(48.5) | 160(27.0) |  |  | 164(27.7) | 245(41.4) | 183(30.9) |  |  |
| 5001–10,000 (US$770–1538) | 118(20.1) | 288(49.2) | 180(30.7) |  |  | 95(16.2) | 253(43.2) | 238(40.6) |  |  |
| > =10,001 (US$1538) | 46(11.7) | 209(53.3) | 137(35.0) |  |  | 48(12.3) | 155(39.5) | 189(48.2) |  |  |
| Parents with medical background |  |  |  | 171.324 | <0.001 |  |  |  | 34.191 | <0.001 |
| Yes | 21(7.6) | 89(32.1) | 167(60.3) |  |  | 33(11.9) | 104(37.6) | 140(50.5) |  |  |
| No | 414(24.8) | 875(52.5) | 378(22.7) |  |  | 396(23.8) | 704(42.2) | 567(34.0) |  |  |
| (b) |  |  |  |  |  |  |  |  |  |  |
| Characteristics | Skills for antibiotic identification(N=2478), N(%)^a^ | | | | | Skills for antibiotic use(N=2478), N(%)^a^ | | | | |
|  | Low | Median | High | χ^2^ / F | P value | Low | Median | High | χ^2^ / F | P value |
| Province |  |  |  | 29.005 | <0.001 |  |  |  | 70.291 | <0.001 |
| Zhejiang | 157(21.9) | 349(48.8) | 210(29.3) |  |  | 124(17.3) | 308(43.0) | 284(39.7) |  |  |
| Guangxi | 272(31.5) | 415(48.1) | 176(20.4) |  |  | 266(30.8) | 311(36.0) | 286(33.2) |  |  |
| Shaanxi | 273(30.4) | 423(47.0) | 203(22.6) |  |  | 302(33.6) | 364(40.5) | 233(25.9) |  |  |
| Gender |  |  |  | 0.790 | 0.674 |  |  |  | 0.578 | 0.749 |
| Male | 351(27.5) | 616(48.4) | 307(24.1) |  |  | 359(28.2) | 511(40.1) | 404(31.7) |  |  |
| Female | 351(29.2) | 571(47.4) | 282(23.4) |  |  | 333(27.7) | 472(39.2) | 399(33.1) |  |  |
| Age(years), Mean(SD) | 4.7(3.7) | 5.4(3.2) | 5.4(3.0) | 11.106 | <0.001 | 5.3(3.6) | 5.3(3.2) | 5.0(3.2) | 2.330 | 0.097 |
| Parents’ highest level of education |  |  |  | 202.501 | <0.001 |  |  |  | 181.897 | <0.001 |
| Middle school and under | 288(47.5) | 231(38.1) | 87(14.4) |  |  | 275(45.4) | 206(34.0) | 125(20.6) |  |  |
| High school | 206(29.4) | 364(51.9) | 131(18.7) |  |  | 224(31.9) | 264(37.7) | 213(30.4) |  |  |
| Junior college and above | 208(17.8) | 592(50.5) | 371(31.7) |  |  | 193(16.5) | 513(43.8) | 465(39.7) |  |  |
| Residence |  |  |  | 34.701 | <0.001 |  |  |  | 17.592 | <0.001 |
| Rural | 369(33.7) | 511(46.7) | 215(19.6) |  |  | 347(31.7) | 432(39.4) | 316(28.9) |  |  |
| Urban | 333(24.1) | 676(48.9) | 374(27.0) |  |  | 345(25.0) | 551(39.8) | 487(35.2) |  |  |
| Average household income (RMB, monthly) |  |  |  | 95.359 | <0.001 |  |  |  | 109.579 | <0.001 |
| < =3000 (US$461) | 203(39.7) | 231(45.2) | 77(15.1) |  |  | 197(38.5) | 192(37.6) | 122(23.9) |  |  |
| 3001–5000 (US$462–769) | 255(32.0) | 367(46.0) | 175(22.0) |  |  | 273(34.3) | 296(37.1) | 228(28.6) |  |  |
| 5001–10,000 (US$770–1538) | 179(24.1) | 366(49.3) | 197(26.6) |  |  | 160(21.6) | 317(42.7) | 265(35.7) |  |  |
| > =10,001 (US$1538) | 65(15.2) | 223(52.1) | 140(32.7) |  |  | 62(14.5) | 178(41.6) | 188(43.9) |  |  |
| Parents with medical background |  |  |  | 130.022 | <0.001 |  |  |  | 31.871 | <0.001 |
| Yes | 28(10.8) | 98(37.8) | 133(51.4) |  |  | 42(16.2) | 97(37.5) | 120(46.3) |  |  |
| No | 674(30.4) | 1089(49.1) | 456(20.5) |  |  | 650(29.3) | 886(39.9) | 683(30.8) |  |  |

SD,standard deviation.

**^a^** Data are N(%) unless otherwise stated.

**Table A3**

**Correlation analysis between skills for antibiotic identification and skills for antibiotic use among parents who self-medicated their children**

**(N=1944)**

|  | Skills for antibiotic identification,N(%)^a^ | | |  |  |
| --- | --- | --- | --- | --- | --- |
| Skills for antibiotic use | Low | Median | High | Gamma | P value |
| Low | 153(35.7) | 201(46.8) | 75(17.5) | 0.232 | <0.001 |
| Medium | 150(18.6) | 433(53.6) | 225(27.8) |  |  |
| High | 132(18.7) | 330(46.7) | 245(34.6) |  |  |

**Table A4**

**Correlation analysis between skills for antibiotic identification and skills for antibiotic use among parents whose children visited hospital(N=2478)**

|  | Skills for antibiotic identification, N(%)^a^ | | |  |  |
| --- | --- | --- | --- | --- | --- |
| Skills for antibiotic use | Low | Median | High | Gamma | P value |
| Low | 305(44.1) | 300(43.3) | 87(12.6) | 0.263 | <0.001 |
| Medium | 203(20.6) | 517(52.6) | 263(26.8) |  |  |
| High | 194(24.1) | 370(46.1) | 239(29.8) |  |  |

**Table A5**

**Sensitivity analysis**

**(a) Parents who self-medicated their children(N=1944) (b) Parents whose children visited hospital(N=2478)**

| **(a)** |  |  |  |
| --- | --- | --- | --- |
|  | Model1 | Model2 | Model3 |
|  | aOR(95% CI) | aOR(95% CI) | aOR(95% CI) |
| Skills for antibiotic identification | 1.00 (0.94,1.06) | 1.09 (1.03,1.16)^**^ | 1.46 (1.32,1.62)^***^ |
| Skills for antibiotic use |  | 0.65 (0.61,0.70)^***^ | 0.98 (0.86,1.11) |
| Skills for antibiotic identification×Skills for antibiotic use |  |  | 0.88 (0.85,0.91)^***^ |
| **(b)** |  |  |  |
|  | OR(95% CI) | OR(95% CI) | OR(95% CI) |
| Skills for antibiotic identification | 1.02 (0.94,1.11) | 1.09 (1.00,1.19) | 1.34 (1.17,1.52)^***^ |
| Skills for antibiotic use |  | 0.78 (0.71,0.86)^***^ | 1.10 (0.92,1.31) |
| Skills for antibiotic identification×Skills for antibiotic use |  |  | 0.89 (0.85,0.94)^***^ |

*< 0.05; **< 0.01; ***< 0.001.

aOR, adjusted odds ratio.
